# Preliminary report of the outcomes and indications of single approach, double-channel core decompression with structural bone support and bone grafting for osteonecrosis of the femoral head

**DOI:** 10.1186/s12891-022-05149-4

**Published:** 2022-03-03

**Authors:** Ju’an Yue, Xiaozhong Guo, Randong Wang, Bing Li, Qiang Sun, Wangyan Liu, Jiao Chen

**Affiliations:** Department of Joint Surgery, Aviation General Hospital, Courtyard 3, Anwai Beiyuan, Chaoyang District, Beijing, China

**Keywords:** Osteonecrosis, Femoral head, Single approach, Double-channel, Core decompression

## Abstract

**Background:**

To report the outcomes of the single approach to double-channel core decompression and bone grafting with structural bone support (SDBS) for osteonecrosis of the femoral head (ONFH) and define the indications.

**Methods:**

One-hundred-and-thirty-nine hips in 96 patients (79 males, 17 females; mean age 37.53±10.31 years, range 14–58 years; mean body mass index 25.15±3.63 kg/m^2^) were retrospectively analysed. The Harris hip score (HHS) was used to assess hip function, and radiographs were used to assess the depth of femoral head collapse. Treatment failure was defined as the performance of total hip arthroplasty (THA). The variables assessed as potential risk factors for surgical failure were: aetiology, Japanese Osteonecrosis Investigation Committee (JIC) type, age, and Association Research Circulation Osseous (ARCO) stage. Complications were recorded.

**Results:**

The mean follow-up time was 29.26±10.02 months. The HHS increased from 79.00±13.61 preoperatively to 82.01±17.29 at final follow-up (*P*=0.041). The average HHS improvement was 3.00±21.86. The combined excellent and good rate at final follow-up (65.6%) was significantly higher than that before surgery (34.5%) (*P*<0.05). On radiographic evaluation, 103 (74.1%) hips remained stable, while 36 (25.9%) had femoral head collapse or aggravation of ONFH. THA was performed in 18 hips. Thus, the overall femoral head survival rate was 87.05% (121/139). The success rate was adversely affected by JIC type, but not by aetiology, age, or ARCO stage. The only complication was a subtrochanteric fracture in one patient.

**Conclusion:**

The SDBS may be an effective method to delay or even terminate the natural progression of ONFH, especially for patients with JIC types B and C1. The SDBS represents a new option for treating early-stage ONFH.

## Background

Osteonecrosis of the femoral head (ONFH) is a local abnormal bone metabolism disease caused by interruption of or damage to the blood supply of the femoral head, resulting in the death of bone cells and marrow components [1, 2]. Without effective diagnosis and treatment, ONFH has a high rate of clinical progression, eventually leading to femoral head collapse and secondary hip osteoarthritis [3]. When the femoral head is severely collapsed, total hip arthroplasty (THA) is required [4]. However, THA is associated with problems such as wear and loosening of the prosthesis over time, potentially requiring several revision surgeries, which causes a great burden on the patient and their family [5]. Therefore, there is a need for a reliable method of joint preservation in patients with early-stage ONFH.

The main purposes of femoral head-preserving surgery for ONFH are to delay the collapse of the femoral head, maintain and restore the normal function of the hip joint, relieve pain, and delay or even avoid total joint replacement. In 1949, Phemister developed a technique in which a core decompression channel is used to place a graft into the necrotic area [6]. A recent modified version of this Phemister technique, called the single approach to double-channel core decompression and bone grafting with structural bone support (SDBS), has shown satisfactory short-term efficacy in treating ONFH [7]. The SDBS consists of two channels. The outer upper channel was mainly the support rod, while the inner lower channel was mainly bone graft. We hypothesized that the strong support of the bracing channel may provide a stable mechanical environment for the survival of the bone graft channel.

This retrospective cohort analysis of patients who had undergone the SDBS aimed to analyse the outcome and investigate the optimal indications of this technique.

## Materials and methods

All procedures performed in studies involving human participants were in accordance with the ethical standards of World Medical Association Declaration of Helsinki Ethical Principles for Medical Research Involving Human Subjects. All methods were carried out in accordance with the Ethics Committee of Aviation General Hospital (No: HK2019-01-04). The Ethics Committee of our hospital approved the use of this material to treat ONFH and approved the retrospective analysis of the results. A total of 103 patients (149 hips) underwent the SDBS for ONFH in a single centre between October 2016 and October 2020. Seven patients (10 hips) were excluded because they were lost to follow-up. The final study cohort comprised 139 hips in 96 patients (79 males and 17 females; mean age 37.53±10.31 years, range 14–58 years; mean body mass index 25.15±3.63 kg/m2). The ONFH was bilateral in 43 patients and unilateral in 53 patients (29 left hips, 24 right hips). The ONFH was caused by prolonged excessive alcohol intake in 32 patients (44 hips), glucocorticoid administration in 42 patients (66 hips), trauma in nine patients (nine hips), and had no clear aetiology in 13 patients (20 hips). All patients were diagnosed in accordance with Chinese guidelines for the diagnosis and treatment of ONFH [8]. The inclusion criteria for this study were: (1) a clear diagnosis of ONFH; (2) radiographic femoral head collapse of less than 2 mm; (3) no history of surgical treatment for ONFH; (4) age <60 years; (5) provision of informed consent for study participation. The exclusion criteria for this study were: (1) weak subjective desire to preserve the hip; (2) inability to tolerate surgery due to other diseases or poor overall condition; (3) active infection or coagulopathy.

Osteonecrosis was classified as Association Research Circulation Osseous (ARCO) stage II in 63 hips, and ARCO stage III in 76 hips. Based on the Japanese Osteonecrosis Investigation Committee (JIC) classification system, the ONFH was classified as type B in 21 hips, type C1 in 54 hips, and type C2 in 64 hips. Characteristics of the patients and hips are listed in Table 1.

**Table 1:** Characteristics of the patients and hips

	Patients	Hips
Male	79	-
Female	17	-
Age (years)	37.53 ± 10.31	-
BMI	25.15±3.63	-
Bilateral	43	86
unilateral	53	53
Alcohol abuse	32	44
Corticosteroid application	42	66
Post-traumatic	9	9
Idiopathic	13	20
ARCOII	-	63
ARCOIII	-	76
JIC B	-	21
JIC C1	-	54
JIC C2	-	64

### Surgical technique

**Fig. 1:**
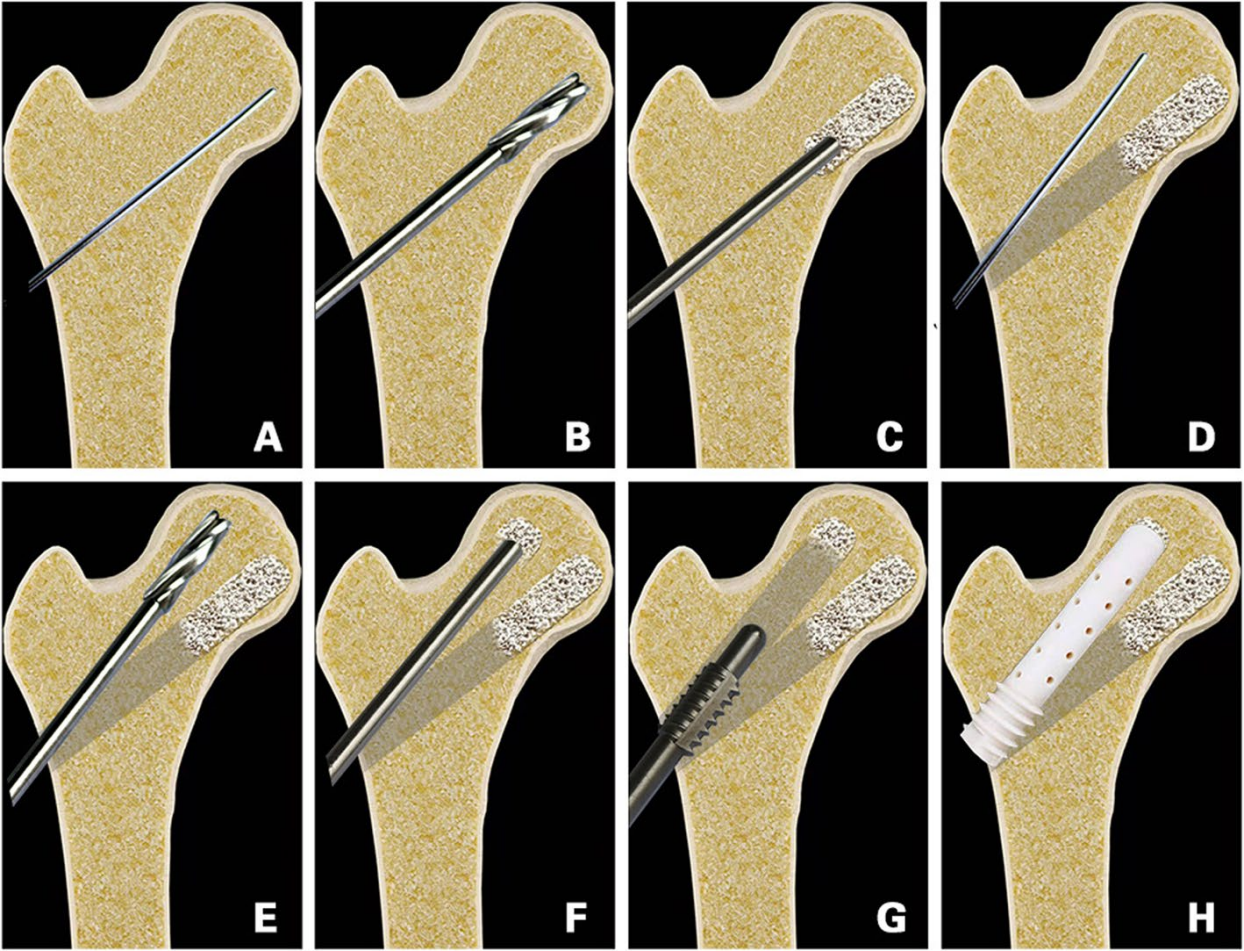
Surgical steps of SDBS

**Fig. 2:**
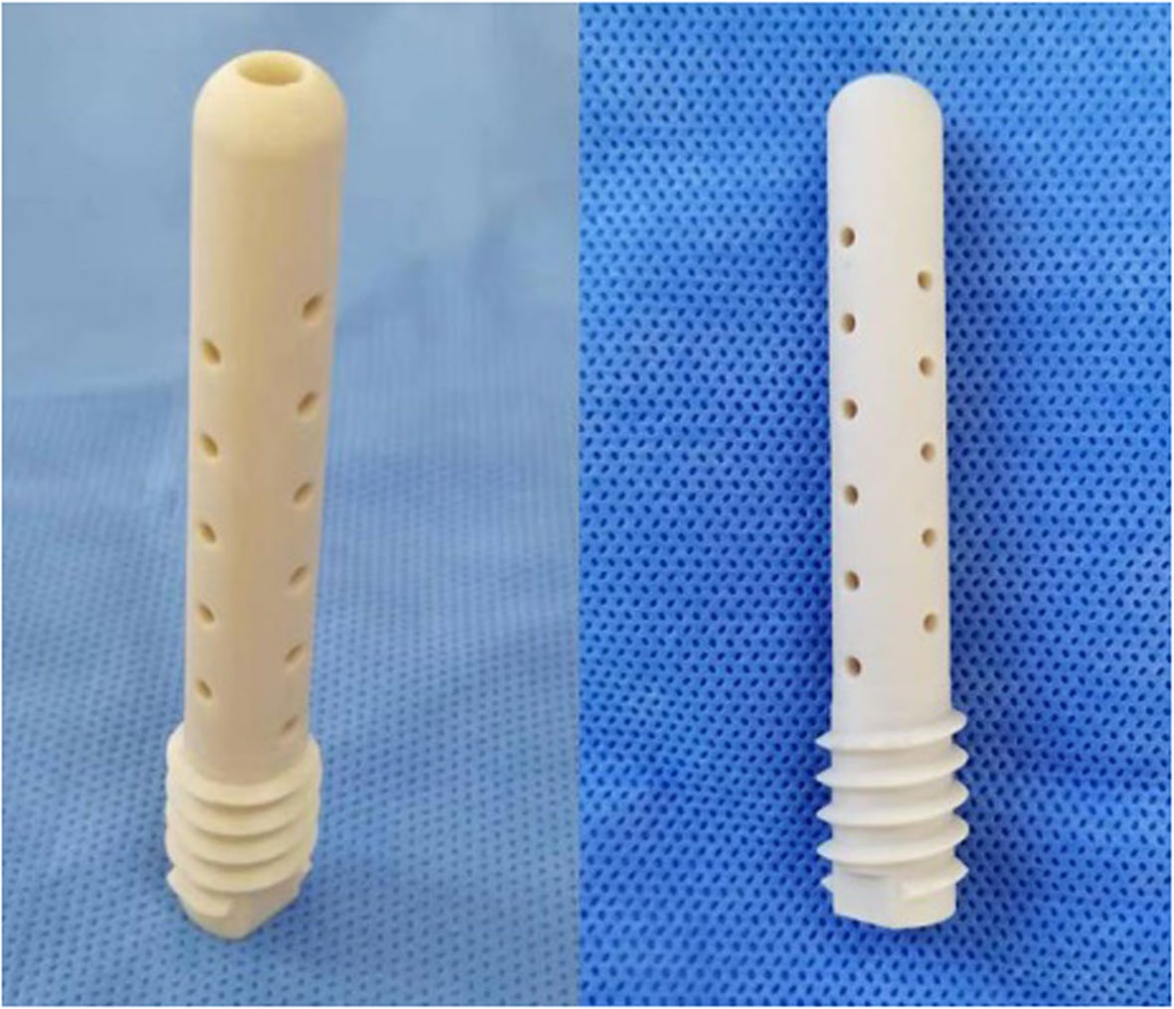
The nano-hydroxyapatite/polyamide-66 (n-HA/PA66) support rod (authorization number: CF180936, hollow cylinder, 10-mm outer diameter, 4-mm inner diameter)

To determine the correct location of the guidewire, tunnel, and bone graft, the surgery was performed under a G-arm X-ray machine. All patients received epidural anesthesia and were fixed on the traction bed. The operation area was routinely disinfected and sterilely draped. After selecting the position of the entrance point, a 2-cm skin incision was made. The first guidewire was drilled into the region below and inside the area of femoral head necrosis (Fig. 1A). A 10-mm bit was then reamed along the guidewire to 3 mm below the cartilage (Fig. 1B). Fresh-frozen allograft particles (7.5 mg) (Shanxi AoRui Biological Material Co., Ltd., Taiyuan, China) were transplanted into the channel from the necrotic area to the normal area (Fig. 1C). After allogeneic bone implantation, the bone graft was compacted with moderate strength. A second guidewire was then introduced through the same entrance point into the outer, top necrotic area (Fig. 1D), and the 10-mm bit was again reamed along the guidewire to 3 mm below the cartilage (Fig. 1E). Fresh-frozen allograft particles (2.5 mg) were transplanted into the second channel (Fig. 1F) and compacted with moderate strength. The length of the upper channel was then measured. The length of the measured channel was consistent with the length of the support rod. After reaming the proximal femur (Fig. 1G), a suitable nano-hydroxyapatite/polyamide 66 (n-HA/PA66) support rod (Sichuan National Nanotechnology Co., Ltd., Chengdu, China; Fig. 2) was inserted into the second channel along the guidewire and fixed by rotation (Fig. 1H). Finally, the wound was irrigated and sutured.

### Postoperative care and follow-up

Postoperatively, cefazolin sodium pentahydrate for injection (1 g) was administered once to prevent infection. Flurbiprofen axetil injection (100 mg twice daily) was routinely given as analgesia for 3 days. All patients participated in a rehabilitation and training program after surgery. After recovery from anaesthesia, the patients began ankle dorsiflexion exercises to prevent deep vein thrombosis without the need for medication. Patients began walking with two crutches from postoperative day 1 and were restricted to partial weight bearing for 6 months. From 6 months postoperatively, patients were permitted to exercise and walk intermittently without crutches. By 1 year postoperatively, patients were fully weight bearing. Postoperative follow-up was carried out at 3, 6, and 12 months postoperatively, and annually thereafter.

### Efficacy assessment

The Harris hip score (HHS) was used to assess the hip function as excellent (HHS ≥ 90), good (HHS 80–89), fair (HHS 70–79), or poor (HHS < 70). At each follow-up visit, all patients underwent CT and radiography (anteroposterior and frog position) of the hip. CT was used to detect subchondral fracture of the femoral head, while radiographs were used to check the depth of the femoral head collapse. Treatment failure was defined as the performance of hip replacement. Other assessed variables included the operation time, intraoperative blood loss volume, and postoperative complications.

### Statistical analysis

SPSS version 22.0 (IBM Corp.; Armonk, NY, USA) was used for statistical analysis. Data are expressed as means ± standard deviations. The paired t test and Wilcoxon test were used to compare the preoperative HHS with the HHS at final follow-up. Rate comparisons were performed using the χ^2^ test. Single risk factor analysis for surgical failure was performed using the Kaplan-Meier method. *P* < 0.05 was considered to indicate statistical significance.

## Results

**Fig. 3:**
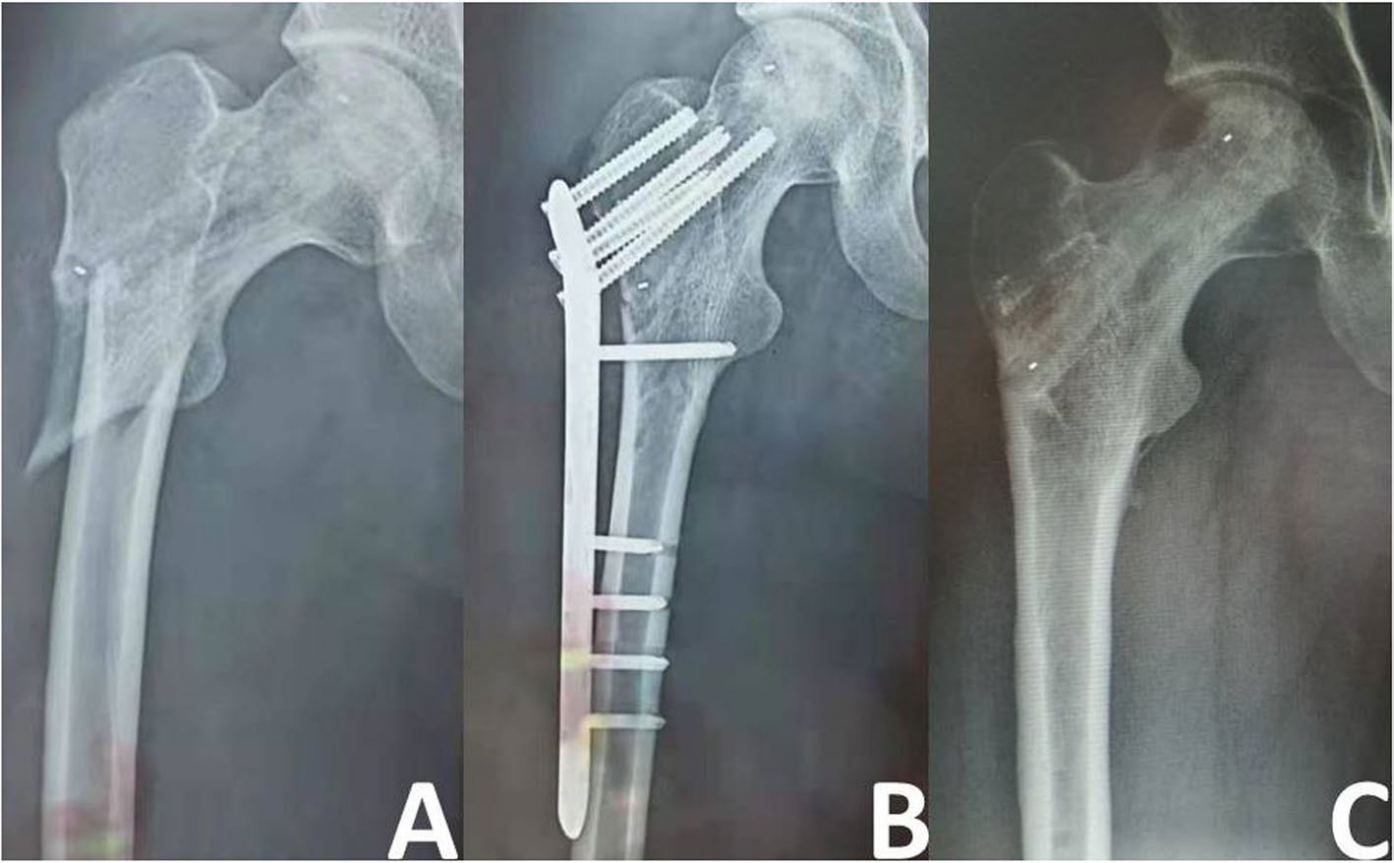
Images of a 28-year-old man with subtrochanteric fracture. **A** Traumatic rotation resulted in subtrochanteric fracture of the femur at 2 weeks after the surgery. **B** Open reduction and internal fixation was performed 3 days after the fracture occurred. **C** At 1 year after fracture fixation, the fracture had healed well and the internal fixation was removed.

The average length of hospital stay was 5.74±0.78 days (range 5–8 days). The average incision length was 3.27±0.22 cm (range 2.0–3.5 cm) and the average intraoperative blood loss volume was 62.02±8.04 ml (range 50–75 ml). The only complication was a subtrochanteric fracture in one patient (Fig. 3); no other complications such as blood vessel or nerve injury, deep vein thrombosis, wound infection, or rejection were observed.

**Fig. 4:**
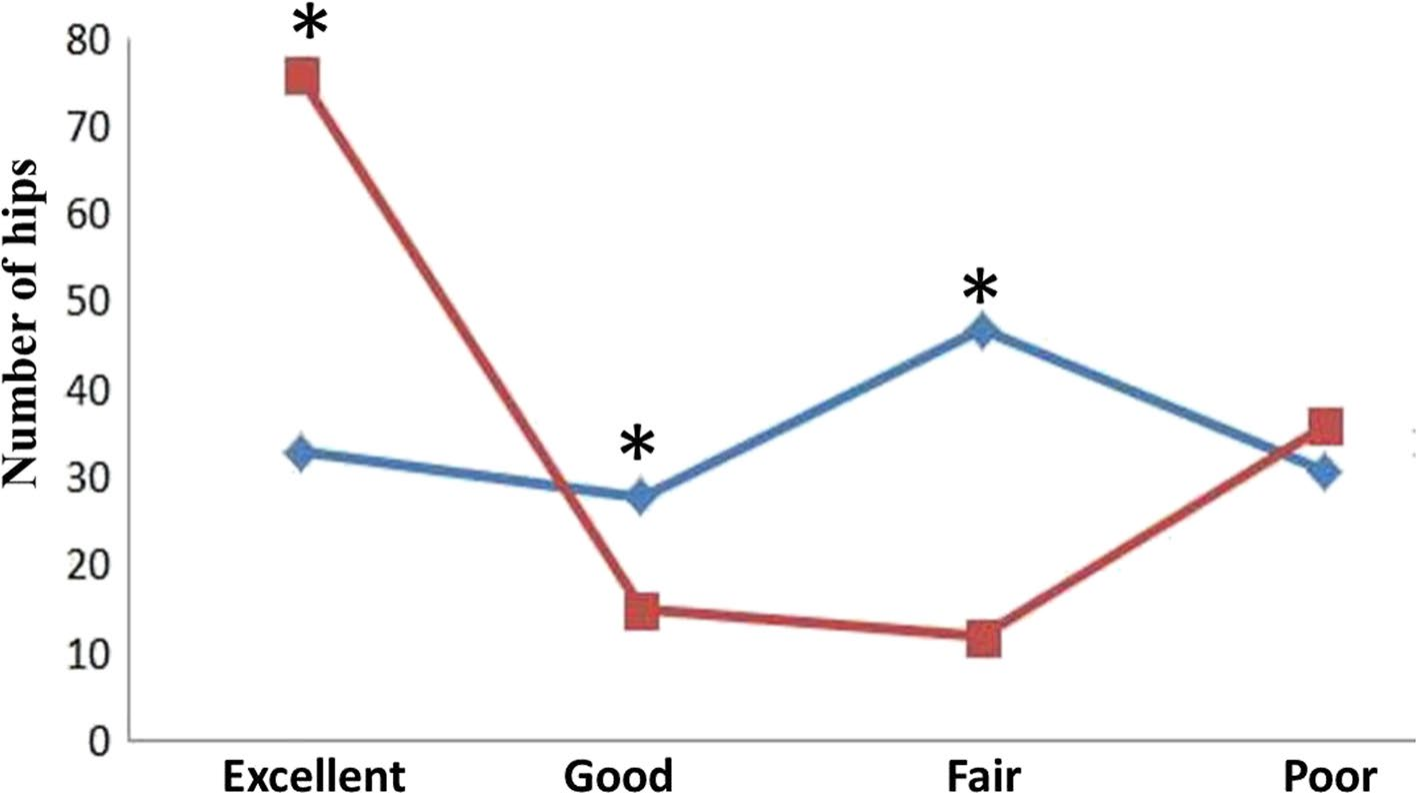
Comparison of the number of hip joints in different stages of hip function. *indicates that composition ratio is statistically significant difference (*P* < 0.05) between the two groups. The blue line represents the number of hip joints at different stages of clinical efficacy before surgery; the red line represents the number of hip joints at different stages of clinical efficacy at the last follow-up.

The mean follow-up time was 29.26±10.02 months. In the overall cohort, the mean HHS increased from 79.00±13.61 preoperatively to 82.01±17.29 at final follow-up (*P*=0.041); the average improvement in the HHS was 3.00±21.86. The details of the HHS changes from preoperatively to final follow-up in subgroups of patients are listed in Table 2. An analysis of patients grouped in accordance with the aetiology of ONFH found that the mean postoperative HHS tended to be improved compared with the mean preoperative HHS in all groups; however, these differences were not significant. An analysis of patients grouped in accordance with JIC type found that the HHS of patients with type C1 was significantly improved from 78.87±12.92 preoperatively to 87.42±14.70 at final follow-up (*P* = 0.007). An analysis of patients grouped by age found that the HHS significantly improved from 78.38±13.15 preoperatively to 84.48±15.602 at final follow-up (*p*=0.003) in those younger than 44 years, but slightly decreased in patients older than 44 years. Although there was no significant difference between the HSS at final follow-up versus preoperatively in patients with ARCO stage II ONFH, the HHS was significantly improved from 73.60±10.955 preoperatively to 81.96±17.83 at final follow-up in patients with ARCO stage III ONFH (*P*<0.05). The combined excellent and good rate at final follow-up (65.6%) was significantly higher than that before surgery (34.5%) (*P*<0.05). Preoperatively and at the last follow-up, the number of hip joints at different stages of clinical efficacy is shown in Fig 4.

**Table 2:** Harris hip score changes in subgroups of patients

	Pre-operative	The last follow-up	Improved score	*P*
Aetiology				
Corticosteroids	79.70±12.51	81.18±18.06	1.48±20.04	0.41
Alcohol abuse	76.51±16.04	82.74±18.07	6.23±26.41	0.056
Traumatic	79.21±9.42	81.62±14.60	2.42±18.20	0.701
Idiopathic	82.07±12.86	83.95±15.03	1.89±17.96	0.644
JIC type				
B	86.38±12.15	87.86±13.58	1.48±18.23	0.714
C1	78.87±12.92	87.42±14.70	8.55±21.94	0.007
C2	76.69±13.97	75.53±18.30	1.16±22.17	0.676
Age (years)				
<44	78.38±13.15	84.48±15.60	6.10±19.96	0.003
>44	80.83±14.90	74.47±20.12	-6.36±24.86	0.133
ARCO stage				
ARCOII	85.52±13.73	82.07±2.11	-3.45±2.83	0.547
ARCO III	73.60±10.955	81.96±17.83	8.36±19.60	0.000
Total	79.00±13.61	82.01±17.29	3.00±21.86	0.041

**Fig. 5:**
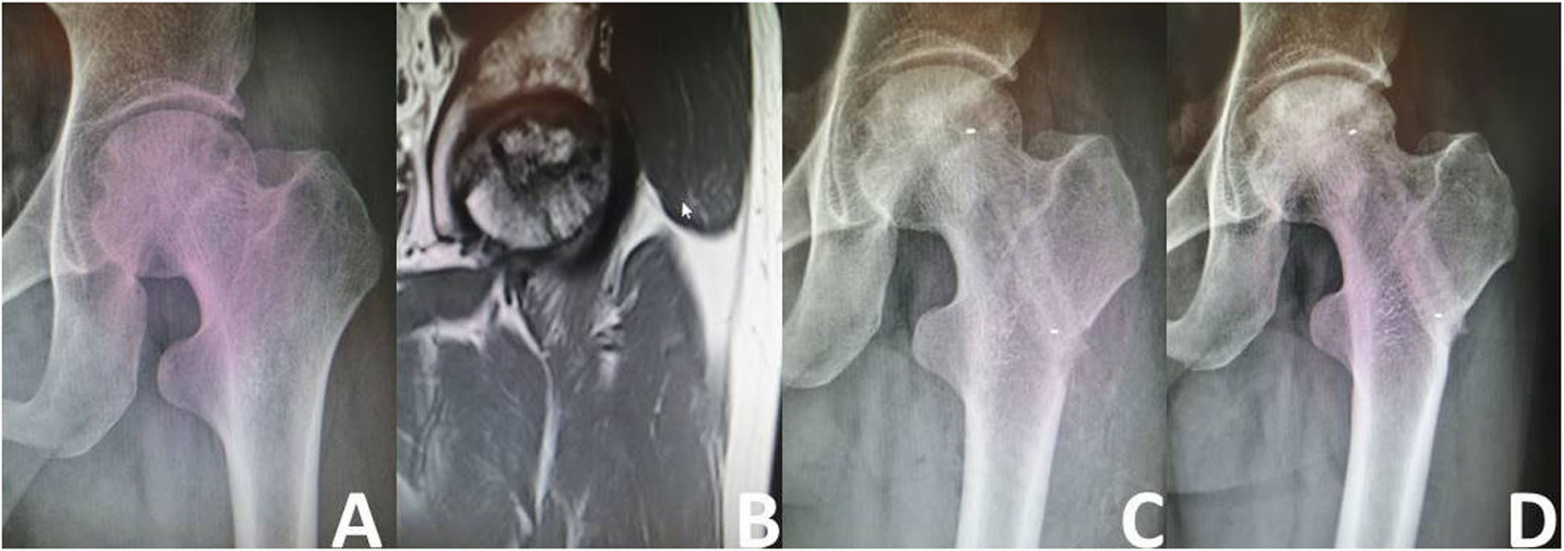
Representative case of stable osteonecrosis of the femoral head. **A** Preoperative radiograph. **B** Preoperative MRI. **C** Radiograph taken on postoperative day 3. **D** Radiographs taken 2 years postoperatively

**Fig. 6:**
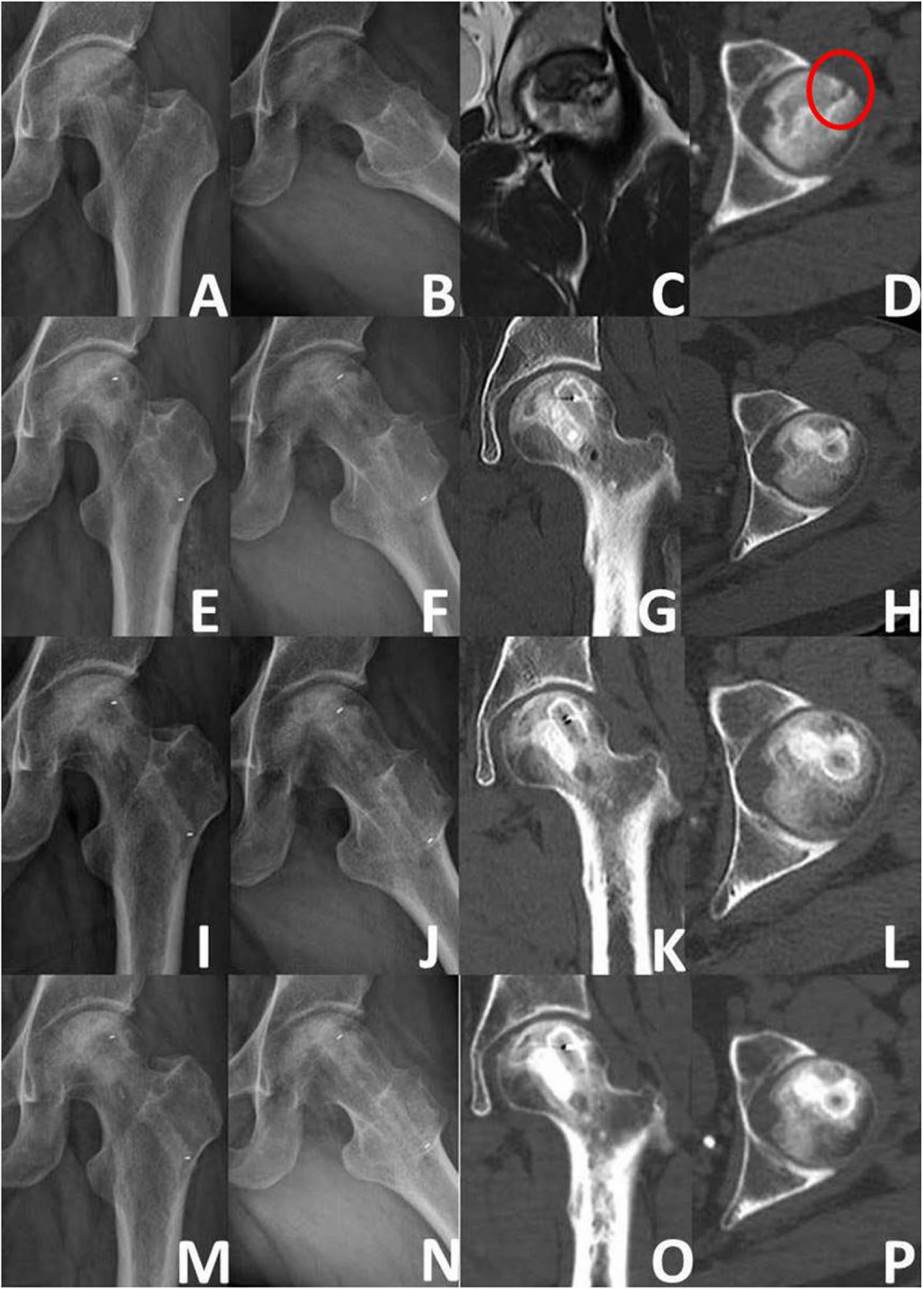
Representative case of ARCO IIIA. **A**, **B** Preoperative radiograph. **C** Preoperative MRI. **D** Preoperative CT (the red circle-subchondral fracture). **E**-**H** 3days post-surgery (X-ray and CT). **I**-**L** 3months post-surgery (X-ray and CT). **M**-**P** 36 months post-surgery (X-ray and CT)

**Fig. 7:**
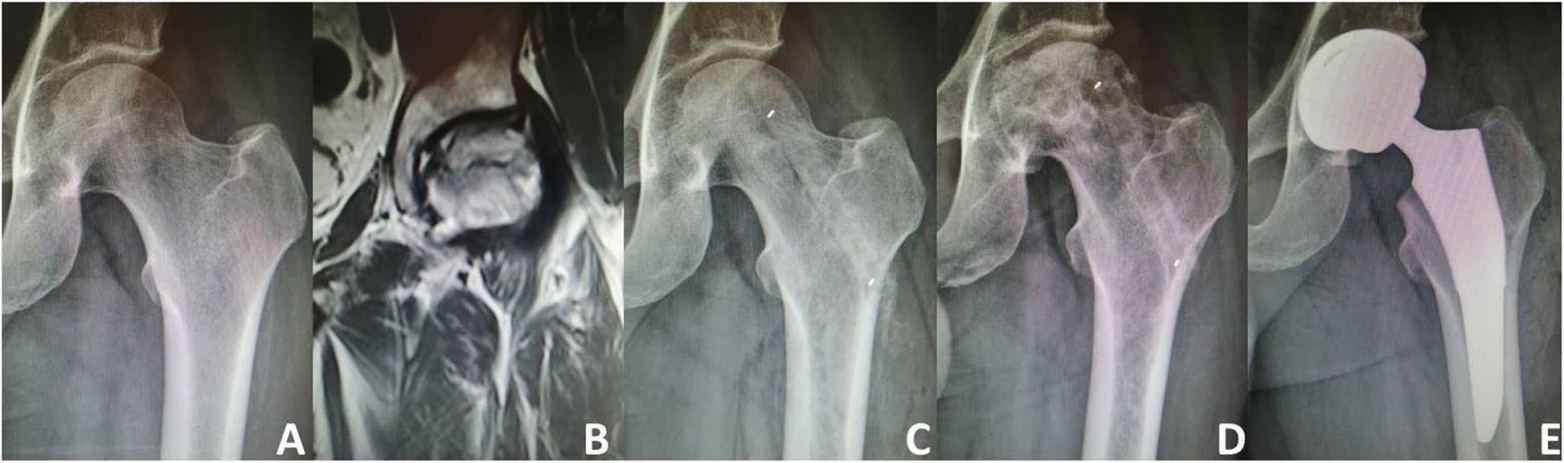
Representative case of treatment failure **A** Preoperative radiograph. **B** Preoperative MRI. **C** Radiograph taken on postoperative day 3. **D** Radiographs taken 1 year after surgery showing severe collapse of the femoral head. **E** The operation failed and a hip replacement was eventually performed

According to the radiographic evaluation, 103 (74.10%) hips remained stable (Figs. 5 and 6), while 36 (25.90%) hips developed collapse of the femoral head or aggravation of ONFH. During the follow-up period, 18 of the 36 hips with radiographically worsened ONFH underwent THA (Fig. 7); the remaining 18 hips did not undergo THA because the hip function was still good. The details of radiographic changes in subgroups of patients are listed in Table 3. There were significant differences in the imaging progression rate of ONFH in accordance with JIC type (*P*=0.001). Radiographic worsening of ONFH was seen in two of 21 hips (8.33%) with JIC type B, eight of 54 hips (14.81%) with JIC type C1, and 26 of 64 hips (40.63%) with JIC type C2; the progression rate of the type C2 group was significantly higher than that of the type C1 and type B groups, while there was no significant difference between the type C1 and type B groups. There were no significant differences in the radiographic changes between patients grouped in accordance with aetiology, age, or ARCO stage (*p*>0.05).

**Table 3:** Radiographic changes in subgroups of patients

	Stable (n)	Worse (n)	Progression rate(%)
Aetiology			
Corticosteroids (*n*=66)	49	17	25.76
Alcohol abuse (*n*=44)	30	14	38.82
Traumatic (*n*=9)	6	3	33.33
Idiopathic (*n*=20)	18	2	10.00
Between the four groups: *P*=0.278			
JIC type			
B (*n*=21)	19^a^	2^a^	9.52
C1 (*n*=54)	46^a^	8^a^	14.81
C2 (*n*=64)	38^b^	26^b^	40.62
Between the three groups: *P*=0.001			
Age (years)			
<44 (*n*=102)	78	24	23.53
>44 (*n*=37)	25	12	32.43
Between the two groups: *P*=0.290			
ARCO stage			
ARCOII (*n*=63)	47	16	25.40
ARCO III (*n*=76)	56	20	26.32
Between the two groups: *P*=0.902			
Total (*n*=139)	103	36	25.90

^a ^No significant difference between the two groups (*P*>0.05)

^b ^Significant difference between the two groups (*P*<0.05)

At final follow-up, 18 hips had undergone THA. Thus, the overall femoral head survival rate was 87.05% (121/139). The single factor analysis of the factors affecting treatment failure are summarized in Table 4. The incidence of THA did not significantly differ between hips grouped in accordance with aetiology (*P*>0.005); of the hips that underwent THA, the aetiology of ONFH was steroid administration in 10 (15.15%) hips, excessive alcohol intake in six (13.64%), trauma in one (11.11%), and idiopathic in one (5%). The incidence of THA significantly differed among groups based on JIC type (*P*<0.05); THA was performed in 15 (23.44%) type C2 hips, three (5.56%) type C1 hips, and no type B hips. The incidence of THA in type C2 hips was significantly higher than that in type C1 and type B hips, while the incidence of THA did not significantly differ between type C1 and type B hips. There were no significant differences in the failure rate among patients grouped in accordance with age and ARCO stage (*P*>0.05). THA was performed in 10 (9.80%) hips in patients younger than 44 years and six (21.62%) hips in patients aged 44 years or older. THA was performed in eight (12.70%) hips classified as ARCO stage II and 10 (13.16%) hips classified as ARCO stage III.

**Table 4:** Single factor analysis of the variables affecting treatment failure

	Successful cases (n)	Failure case (n)	χ2	Log-rank P
Aetiology			1.349	0.717
Corticosteroids (*n*=66)	56	10		
Alcohol abuse (*n*=44)	38	6		
Traumatic (*n*=9)	8	1		
Idiopathic (*n*=20)	19	1		
Between the four groups: *P*=0.767				
JIC type			14.256	0.001
B (*n*=21)	21^a^	0^a^		
C1 (*n*=54)	51^a^	3^a^		
C2 (*n*=64)	49^b^	15^b^		
Between the three groups: *P*=0.003				
Age (years)			3.815	0.051
<44 (*n*=102)	92	10		
≥44 (*n*=37)	29	8		
Between the two groups: *P*=0.122				
ARCO stage			0.063	0.802
ARCOII (*n*=63)	55	8	12.70	
ARCO III (*n*=76)	66	10	13.6	
Between the two groups: *P*=0.936				
Total (*n*=139)	121	18		

^a ^No significant difference between the two groups (*P*>0.05)

^b ^Significant difference between the two groups (*P*<0.05)

**Fig. 8:**
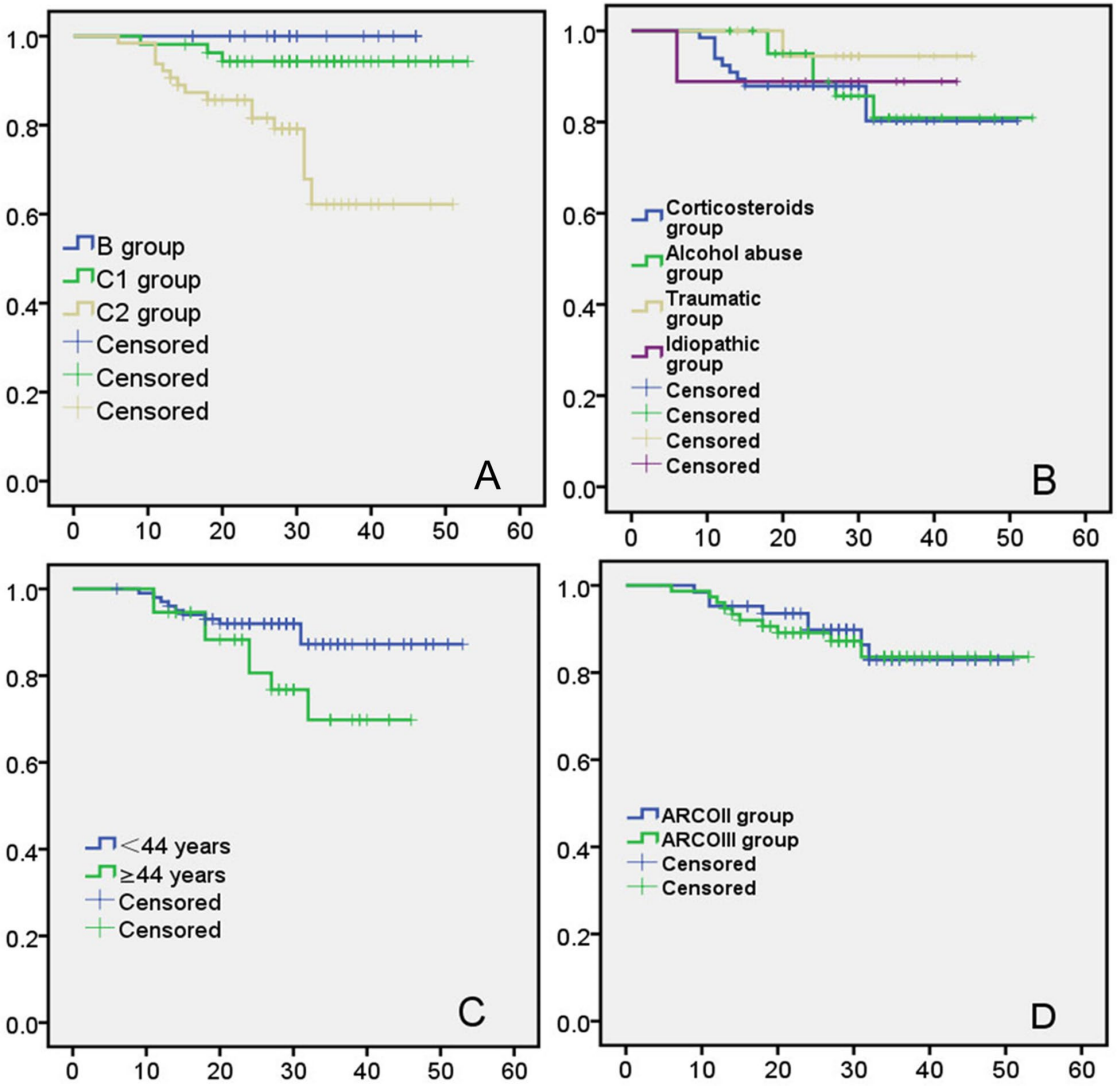
Kaplan-Meier survival curve (The endpoint is revision to THA): **A** stratified according to JIC type; **B** stratified according to aetiology; **C** stratified according to age; **D** stratified according to ARCO stage

Univariate analysis showed that the femoral head survival rate was significantly affected by the JIC type (χ^2^=14.26, *P*=0.001) (Fig. 8A), but not the aetiology (Fig. 8B), age (Fig. 8C), or ARCO stage (Fig. 8D) (*P*>0.05).

## Discussion

The SDBS is an improvement on the Phemister technique [7]. The Phemister technique uses a hole to decompress the necrotic area of the femoral head, and then fills the hole with bone graft material to facilitate the repair of the necrotic area [9–11]. The SDBS establishes two channels in the necrotic area for decompression, support, and allograft placement: an outer and upper channel, and an anterior and medial channel. Compared with the Phemister technique, the SDBS is thought to increase the support strength and provide sufficient allogeneic cancellous bone in the necrotic areas.

**Fig. 9:**
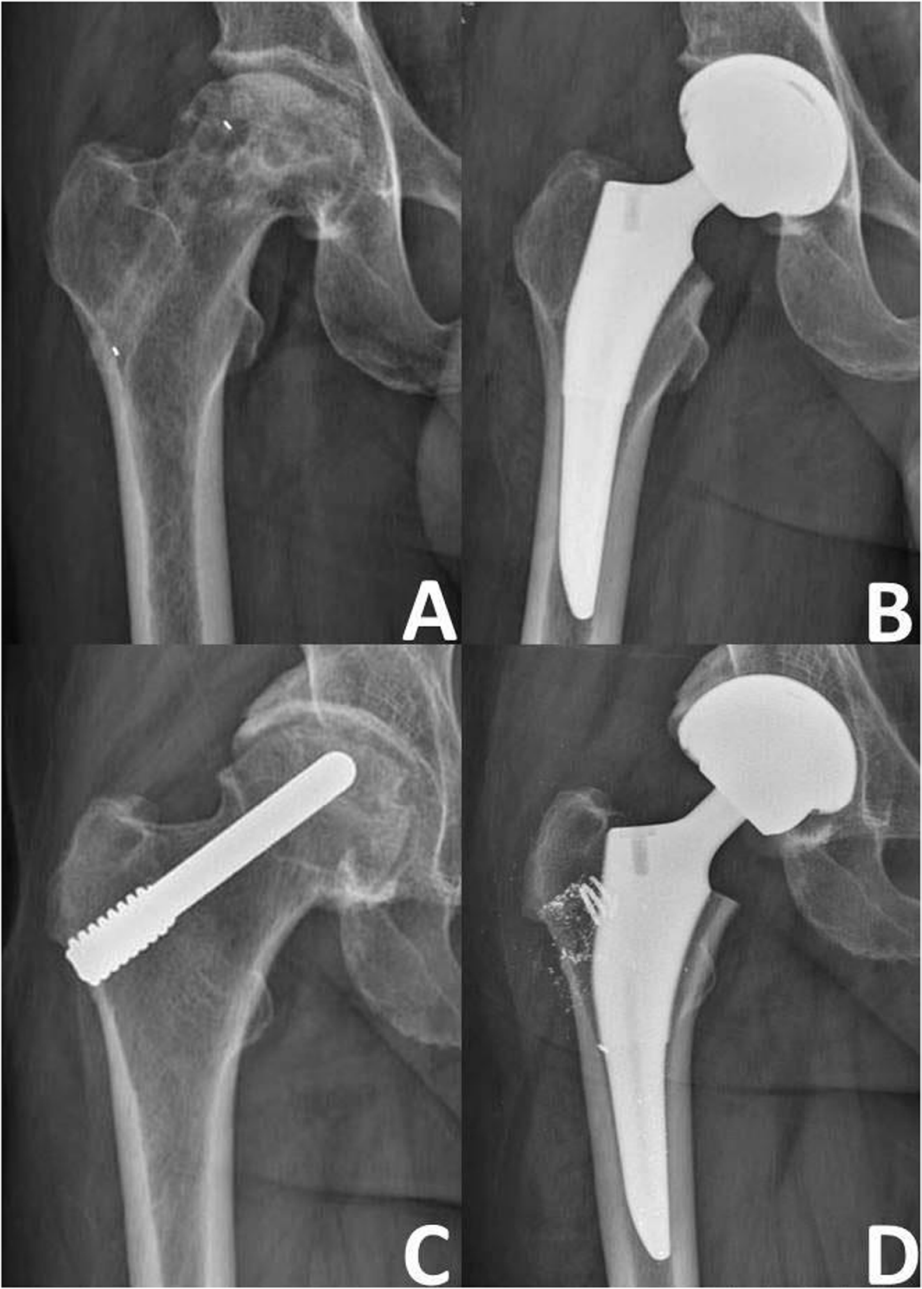
Failure cases with different support rods. **A** Failure case with a n-HA/PA66 rod. **B** Failure case with a n-HA/PA rod converted to THA. **C** Failure case with a porous tantalum rod. **D** Failure case with a porous tantalum rod converted to THA (metal debris can be seen around the prosthesis)

In the present study, a n-HA/PA66 support rod composed of nanometer HA crystal particles evenly dispersed in PA66 was used. Although HA has been successfully used in the treatment of bone injuries, it is brittle and has poor compressive resistance, and cannot be applied in a load-bearing environment [11]. The compressive strength of n-HA/PA66 made by the fusion of HA and PA66 is as high as 13.2 MPa, which is close to the compressive strength of human cancellous bone. This is because when HA and PA66 are under pressure, the relatively hard HA prevents the relatively soft PA from bending and compression, and the toughness of PA makes up for the brittleness of HA [12, 13]. The n-HA/PA66 has a porosity of about 70% and pore size of 200–500 μm, and the porosity is interconnected to create a good three-dimensional space for the introduction of blood vessels [14]. Previous studies have shown that n-HA/PA66 implanted into the skull defect of rats resulted in healing of the skull defect within 8 weeks [15], and that n-HA/PA66 implanted into a massive mandibular defect in rabbits resulted in the presence of active osteoblasts and callus formation in the pores of the scaffold at 4 weeks and complete healing of the bone defect at 24 weeks [16]. These previous findings suggest that n-HA/PA66 has good bone conductivity and promotes bone regeneration. Through clinical observation we found that, when THA is needed (Fig. 9A), a n-HA/PA66 support rod can be easily cut off without leaving any debris (Fig. 9B) or requiring a longer operation time. In contrast, trabecular metal rods such as porous tantalum (Fig. 9C) are difficult to cut off and remove, which increases the operation time and leaves a small amount of metal debris in the soft tissue (Fig. 9D). The effect of metal debris on the outcome of THA needs further study.

Many studies have reported the outcomes of ONFH treatment using different bone grafts and structural supports. Free vascularized fibular grafts for post-traumatic ONFH reportedly resulted in a survival rate of 64% and average HHS of 77.3±24.57 points after a mean follow-up of 10.9 years [17]. A long-term comparative study of non-vascularized autologous versus allogeneic fibular grafts for ONFH found no significant differences in the HHS (80.3±14.5 and 82.4±13.6 respectively) and survival rate (84.1 and 86% respectively) [18]. After a mean follow-up of 42 months, the survival rate of a porous tantalum implant combined with bone grafting to treat ONFH was only 52.9%, which shows that this is not a viable option for treating ONFH [19]. Core decompression in combination with a n-HA/PA66 rod and a porous bioglass bone graft for treating ONFH resulted in a clinical failure rate of 23.68% and an improvement in the HHS of 27.19±2.79 after a mean follow-up of 21.78±8.46 months [20]. The treatment of early-stage ONFH with a n-HA/PA66 rod resulted in a rate of excellent and good short-term outcomes of 76% and a short-term success rate of 94.52% [7]. In the present study cohort treated with SDBS, the HHS reached 82.01±17.29 and the survival rate was 87.05% after 29.26±10.02 months of follow-up.

The JIC classification system for ONFH is based on the acetabulum site corresponding to the femoral head lesions [21]. Small lesions located medially or centrally, such as types A and B, are much less likely to progress to collapse than lesions that occupy most of the weightbearing area, such as types C1 and C2 [22]. In type C2, the osteonecrosis extends to the lateral wall of the femoral head, resulting in the loss of structural integrity. In our study, the postoperative imaging progression rate of patients with type C2 ONFH was as high as 40.62% and was significantly higher than that of patients with types C1 and B. The rate of surgical failure was also significantly higher in patients with type C2 than in those with type C1 and B. There have been no reports that sex, age, BMI, or associated factors are related to the progression of ONFH [22]. However, the progressive necrosis and collapse of the femoral head is reportedly worse in patients with type C2 ONFH compared with other JIC types [19]. Furthermore, after ONFH treatment with non-vascularized bone grafts, patients with type C2 have worse hip function than those with type C1 [23]. By analysing the factors that influenced the surgical success rate, we found that the JIC classification was helpful in predicting the outcome. The success rate of the SDBS for type C2 ONFH was relatively low.

The present study has some limitations. First, many factors may affect the success rate of the SDBS. As we only analysed four factors in this study, other possible influencing factors need to be studied. Second, the follow-up time was relatively short; long-term follow-up is warranted. Third, there was no control group in this single-group retrospective cohort analysis. Fourth, further study is warranted to determine whether this operation affects the blood circulation of the femoral head.

## Conclusion

The SDBS may be an effective method to delay or even terminate the natural progression of ONFH, especially for patients with JIC types B and C1. This operation is minimally invasive, enables a quick postoperative recovery, and has no donor site complications. The SDBS represents a new option for treating early-stage ONFH.

## Data Availability

All data and materials used to support the findings of this study are included within the article.

## Abbreviations

ONFH: Osteonecrosis of the femoral head
THA: Total hip arthroplasty
SDBS: Single approach to double-channel core decompression and bone grafting with structural bone support
ARCO: Association Research Circulation Osseous
JIC: Japanese Osteonecrosis Investigation Committee
HHS: Harris hip score
n-HA/PA66: Nano – hydroxyapatite/polyamide 66
